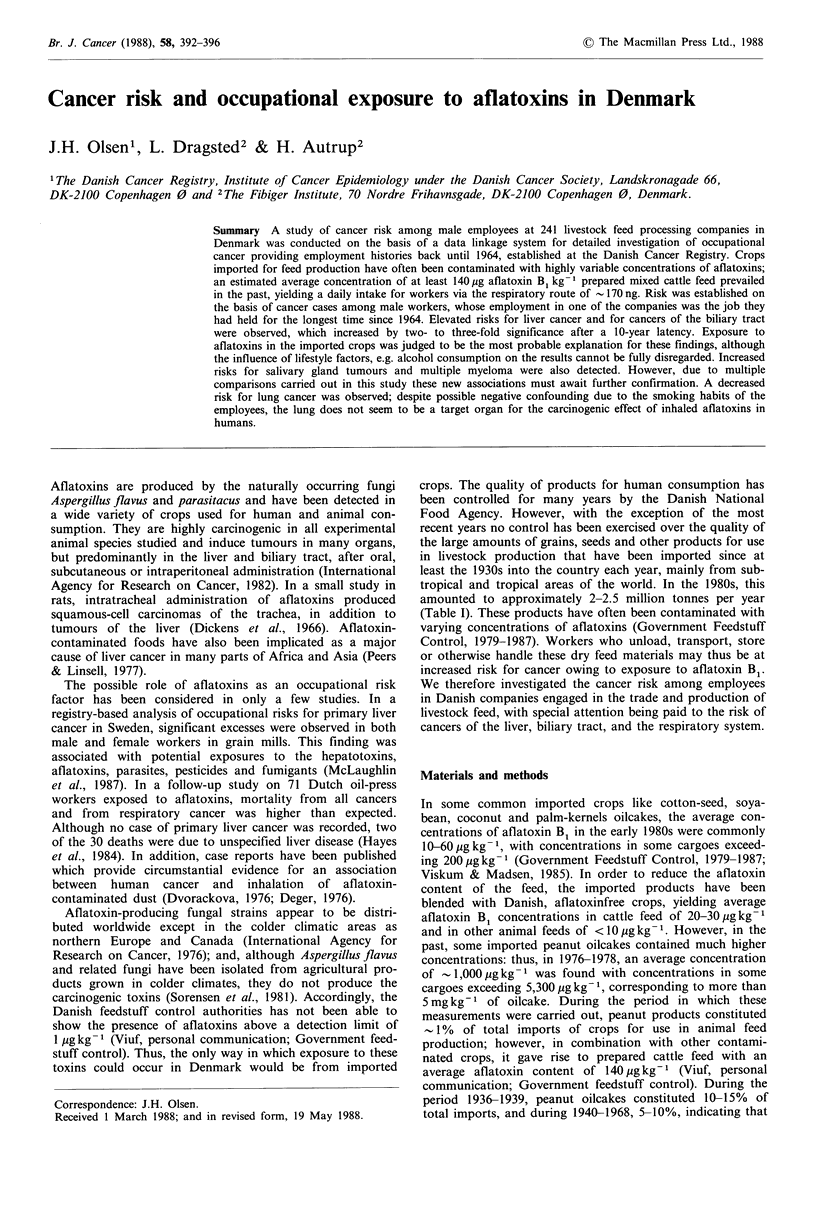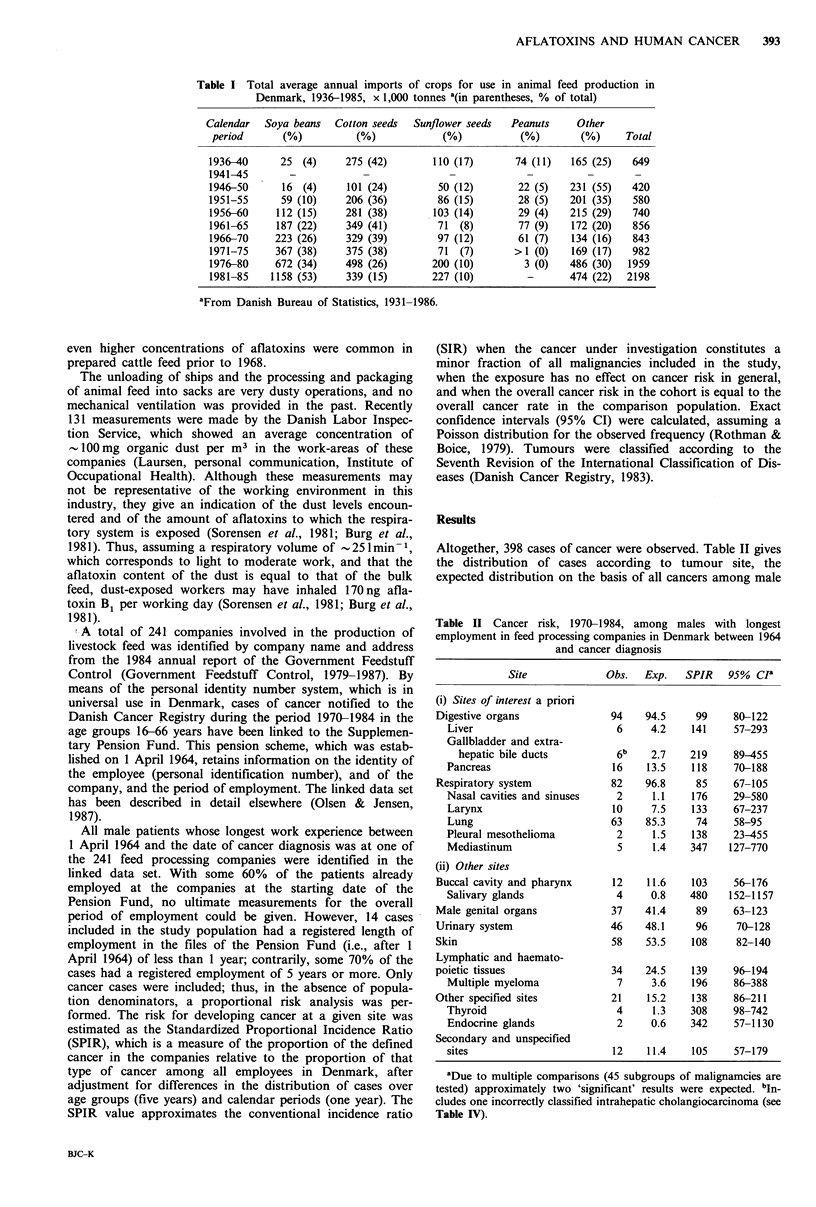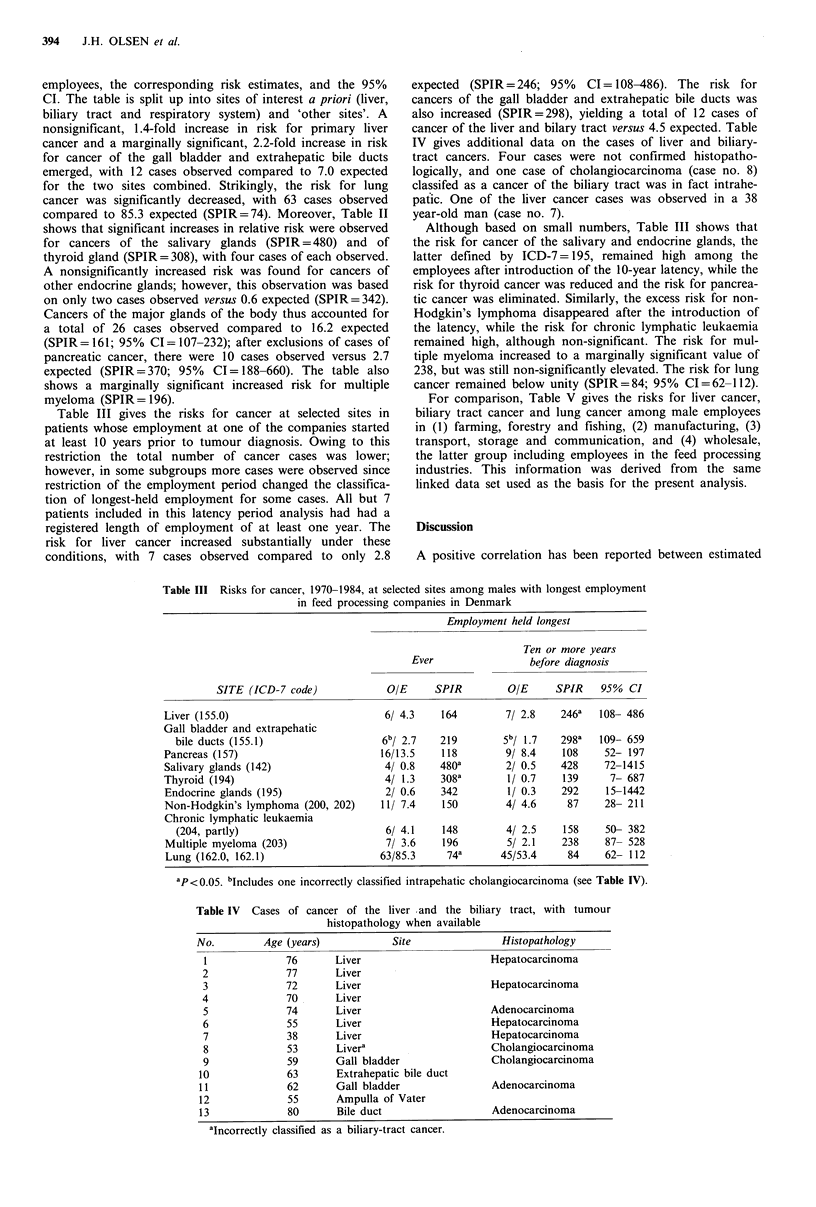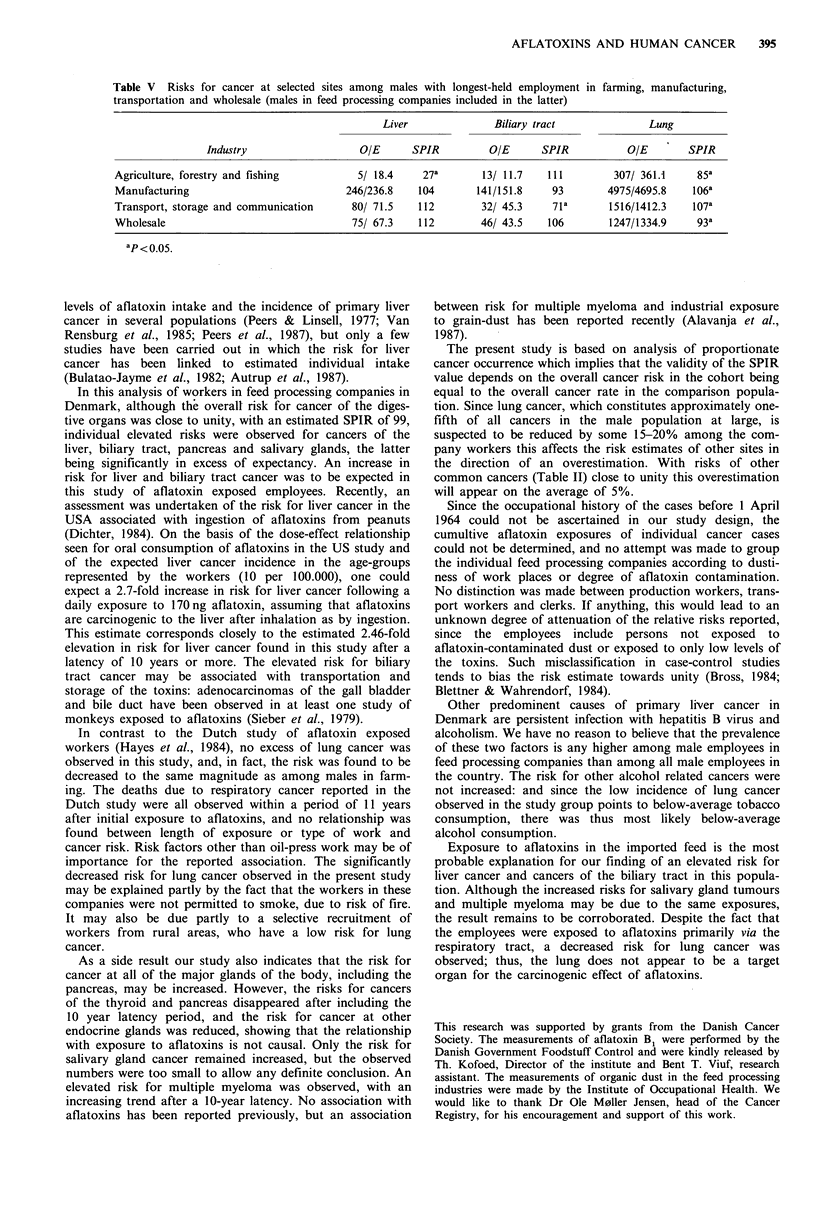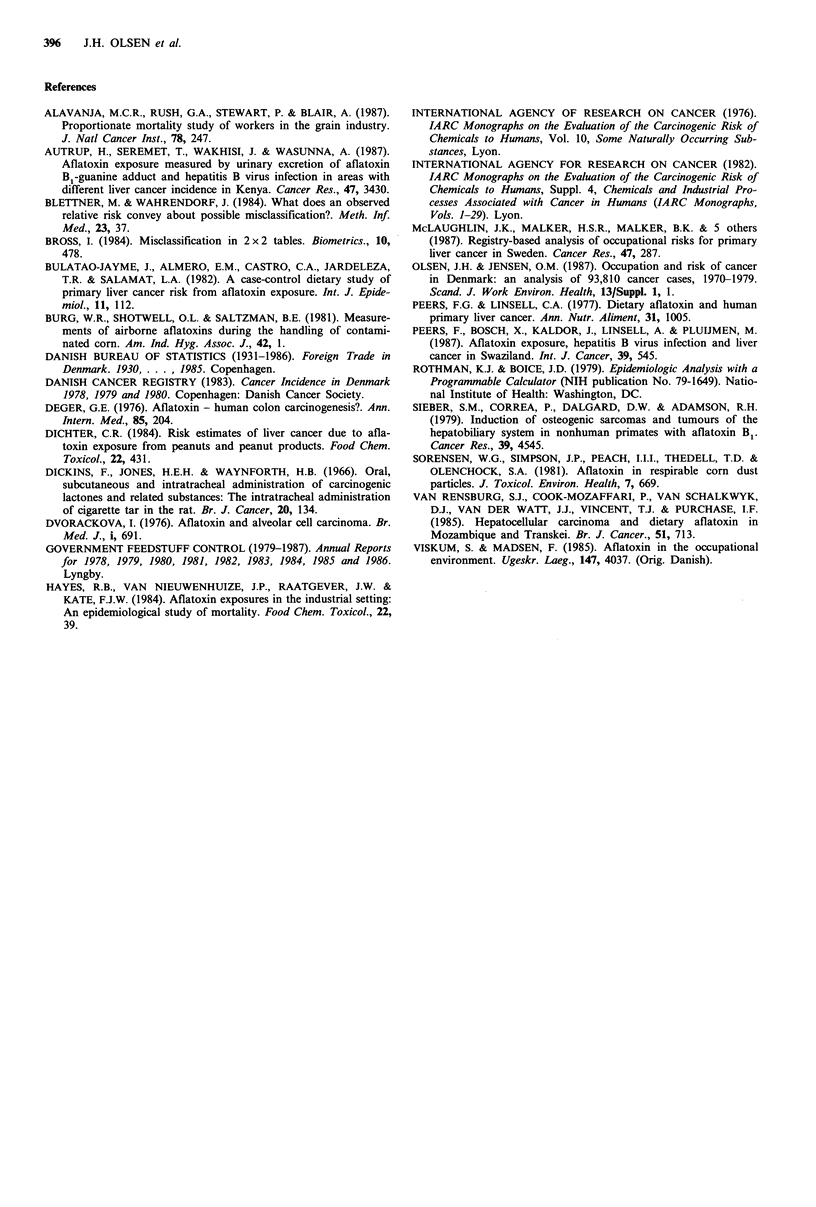# Cancer risk and occupational exposure to aflatoxins in Denmark.

**DOI:** 10.1038/bjc.1988.226

**Published:** 1988-09

**Authors:** J. H. Olsen, L. Dragsted, H. Autrup

**Affiliations:** Danish Cancer Registry, Institute of Cancer Epidemiology, Copenhagen.

## Abstract

A study of cancer risk among male employees at 241 livestock feed processing companies in Denmark was conducted on the basis of a data linkage system for detailed investigation of occupational cancer providing employment histories back until 1964, established at the Danish Cancer Registry. Crops imported for feed production have often been contaminated with highly variable concentrations of aflatoxins; an estimated average concentration of at least 140 micrograms aflatoxin B1 kg-1 prepared mixed cattle feed prevailed in the past, yielding a daily intake for workers via the respiratory route of approximately 170 ng. Risk was established on the basis of cancer cases among male workers, whose employment in one of the companies was the job they had held for the longest time since 1964. Elevated risks for liver cancer and for cancers of the biliary tract were observed, which increased by two- to three-fold significance after a 10-year latency. Exposure to aflatoxins in the imported crops was judged to be the most probable explanation for these findings, although the influence of lifestyle factors, e.g. alcohol consumption on the results cannot be fully disregarded. Increased risks for salivary gland tumours and multiple myeloma were also detected. However, due to multiple comparisons carried out in this study these new associations must await further confirmation. A decreased risk for lung cancer was observed; despite possible negative confounding due to the smoking habits of the employees, the lung does not seem to be a target organ for the carcinogenic effect of inhaled aflatoxins in humans.


### Cancer risk and occupational exposure to aflatoxins in Denmark

J.H. Olsen[1], L. Dragsted[2] & H. Autrup[2]

[1]*The Danish Cancer Registry, Institute of Cancer Epidemiology under the Danish Cancer Society, Landskronagade 66, DK-2100 Copenhagen Ø and* [2]*The Fibiger Institute, 70 Nordre Frihavnsgade, DK-2100 Copenhagen Ø, Denmark.*

**Summary** A study of cancer risk among male employees at 241 livestock feed processing companies in Denmark was conducted on the basis of a data linkage system for detailed investigation of occupational cancer providing employment histories back until 1964, established at the Danish Cancer Registry. Crops imported for feed production have often been contaminated with highly variable concentrations of aflatoxins; an estimated average concentration of at least 140 $\mu$g aflatoxin $B_1$ kg$^{-1}$ prepared mixed cattle feed prevailed in the past, yielding a daily intake for workers via the respiratory route of $\sim$170 ng. Risk was established on the basis of cancer cases among male workers, whose employment in one of the companies was the job they had held for the longest time since 1964. Elevated risks for liver cancer and for cancers of the biliary tract were observed, which increased by two- to three-fold significance after a 10-year latency. Exposure to aflatoxins in the imported crops was judged to be the most probable explanation for these findings, although the influence of lifestyle factors, e.g. alcohol consumption on the results cannot be fully disregarded. Increased risks for salivary gland tumours and multiple myeloma were also detected. However, due to multiple comparisons carried out in this study these new associations must await further confirmation. A decreased risk for lung cancer was observed; despite possible negative confounding due to the smoking habits of the employees, the lung does not seem to be a target organ for the carcinogenic effect of inhaled aflatoxins in humans.

Aflatoxins are produced by the naturally occurring fungi *Aspergillus flavus* and *parasitacus* and have been detected in a wide variety of crops used for human and animal consumption. They are highly carcinogenic in all experimental animal species studied and induce tumours in many organs, but predominantly in the liver and biliary tract, after oral, subcutaneous or intraperitoneal administration (International Agency for Research on Cancer, 1982). In a small study in rats, intratracheal administration of aflatoxins produced squamous-cell carcinomas of the trachea, in addition to tumours of the liver (Dickens *et al.*, 1966). Aflatoxin-contaminated foods have also been implicated as a major cause of liver cancer in many parts of Africa and Asia (Peers & Linsell, 1977).

The possible role of aflatoxins as an occupational risk factor has been considered in only a few studies. In a registry-based analysis of occupational risks for primary liver cancer in Sweden, significant excesses were observed in both male and female workers in grain mills. This finding was associated with potential exposures to the hepatotoxins, aflatoxins, parasites, pesticides and fumigants (McLaughlin *et al.*, 1987). In a follow-up study on 71 Dutch oil-press workers exposed to aflatoxins, mortality from all cancers and from respiratory cancer was higher than expected. Although no case of primary liver cancer was recorded, two of the 30 deaths were due to unspecified liver disease (Hayes *et al.*, 1984). In addition, case reports have been published which provide circumstantial evidence for an association between human cancer and inhalation of aflatoxin-contaminated dust (Dvorackova, 1976; Deger, 1976).

Aflatoxin-producing fungal strains appear to be distributed worldwide except in the colder climatic areas as northern Europe and Canada (International Agency for Research on Cancer, 1976); and, although *Aspergillus flavus* and related fungi have been isolated from agricultural products grown in colder climates, they do not produce the carcinogenic toxins (Sorensen *et al.*, 1981). Accordingly, the Danish feedstuff control authorities has not been able to show the presence of aflatoxins above a detection limit of 1 $\mu$g kg$^{-1}$ (Viuf, personal communication; Government feedstuff control). Thus, the only way in which exposure to these toxins could occur in Denmark would be from imported crops. The quality of products for human consumption has been controlled for many years by the Danish National Food Agency. However, with the exception of the most recent years no control has been exercised over the quality of the large amounts of grains, seeds and other products for use in livestock production that have been imported since at least the 1930s into the country each year, mainly from subtropical and tropical areas of the world. In the 1980s, this amounted to approximately 2–2.5 million tonnes per year (Table I). These products have often been contaminated with varying concentrations of aflatoxins (Government Feedstuff Control, 1979–1987). Workers who unload, transport, store or otherwise handle these dry feed materials may thus be at increased risk for cancer owing to exposure to aflatoxin $B_1$. We therefore investigated the cancer risk among employees in Danish companies engaged in the trade and production of livestock feed, with special attention being paid to the risk of cancers of the liver, biliary tract, and the respiratory system.

### Materials and methods

In some common imported crops like cotton-seed, soyabean, coconut and palm-kernels oilcakes, the average concentrations of aflatoxin $B_1$ in the early 1980s were commonly 10–60 $\mu$g kg$^{-1}$, with concentrations in some cargoes exceeding 200 $\mu$g kg$^{-1}$ (Government Feedstuff Control, 1979–1987; Viskum & Madsen, 1985). In order to reduce the aflatoxin content of the feed, the imported products have been blended with Danish, aflatoxinfree crops, yielding average aflatoxin $B_1$ concentrations in cattle feed of 20–30 $\mu$g kg$^{-1}$ and in other animal feeds of $<10$ $\mu$g kg$^{-1}$. However, in the past, some imported peanut oilcakes contained much higher concentrations: thus, in 1976–1978, an average concentration of $\sim$1,000 $\mu$g kg$^{-1}$ was found with concentrations in some cargoes exceeding 5,300 $\mu$g kg$^{-1}$, corresponding to more than 5 mg kg$^{-1}$ of oilcake. During the period in which these measurements were carried out, peanut products constituted $\sim$1% of total imports of crops for use in animal feed production; however, in combination with other contaminated crops, it gave rise to prepared cattle feed with an average aflatoxin content of 140 $\mu$g kg$^{-1}$ (Viuf, personal communication; Government feedstuff control). During the period 1936–1939, peanut oilcakes constituted 10–15% of total imports, and during 1940–1968, 5–10%, indicating that

Correspondence: J.H. Olsen.
Received 1 March 1988; and in revised form, 19 May 1988.

**Table I** Total average annual imports of crops for use in animal feed production in Denmark, 1936–1985, × 1,000 tonnes [a](in parentheses, % of total)

| *Calendar period* | *Soya beans* (%) | *Cotton seeds* (%) | *Sunflower seeds* (%) | *Peanuts* (%) | *Other* (%) | *Total* |
|---|---|---|---|---|---|---|
| 1936–40 | 25 (4) | 275 (42) | 110 (17) | 74 (11) | 165 (25) | 649 |
| 1941–45 | – | – | – | – | – | – |
| 1946–50 | 16 (4) | 101 (24) | 50 (12) | 22 (5) | 231 (55) | 420 |
| 1951–55 | 59 (10) | 206 (36) | 86 (15) | 28 (5) | 201 (35) | 580 |
| 1956–60 | 112 (15) | 281 (38) | 103 (14) | 29 (4) | 215 (29) | 740 |
| 1961–65 | 187 (22) | 349 (41) | 71 (8) | 77 (9) | 172 (20) | 856 |
| 1966–70 | 223 (26) | 329 (39) | 97 (12) | 61 (7) | 134 (16) | 843 |
| 1971–75 | 367 (38) | 375 (38) | 71 (7) | >1 (0) | 169 (17) | 982 |
| 1976–80 | 672 (34) | 498 (26) | 200 (10) | 3 (0) | 486 (30) | 1959 |
| 1981–85 | 1158 (53) | 339 (15) | 227 (10) | – | 474 (22) | 2198 |

[a]From Danish Bureau of Statistics, 1931–1986.

even higher concentrations of aflatoxins were common in prepared cattle feed prior to 1968.

The unloading of ships and the processing and packaging of animal feed into sacks are very dusty operations, and no mechanical ventilation was provided in the past. Recently 131 measurements were made by the Danish Labor Inspection Service, which showed an average concentration of $\sim 100$ mg organic dust per $m^3$ in the work-areas of these companies (Laursen, personal communication, Institute of Occupational Health). Although these measurements may not be representative of the working environment in this industry, they give an indication of the dust levels encountered and of the amount of aflatoxins to which the respiratory system is exposed (Sorensen *et al.*, 1981; Burg *et al.*, 1981). Thus, assuming a respiratory volume of $\sim 25 \, l \, min^{-1}$, which corresponds to light to moderate work, and that the aflatoxin content of the dust is equal to that of the bulk feed, dust-exposed workers may have inhaled 170 ng aflatoxin $B_1$ per working day (Sorensen *et al.*, 1981; Burg *et al.*, 1981).

A total of 241 companies involved in the production of livestock feed was identified by company name and address from the 1984 annual report of the Government Feedstuff Control (Government Feedstuff Control, 1979–1987). By means of the personal identity number system, which is in universal use in Denmark, cases of cancer notified to the Danish Cancer Registry during the period 1970–1984 in the age groups 16–66 years have been linked to the Supplementary Pension Fund. This pension scheme, which was established on 1 April 1964, retains information on the identity of the employee (personal identification number), and of the company, and the period of employment. The linked data set has been described in detail elsewhere (Olsen & Jensen, 1987).

All male patients whose longest work experience between 1 April 1964 and the date of cancer diagnosis was at one of the 241 feed processing companies were identified in the linked data set. With some 60% of the patients already employed at the companies at the starting date of the Pension Fund, no ultimate measurements for the overall period of employment could be given. However, 14 cases included in the study population had a registered length of employment in the files of the Pension Fund (i.e., after 1 April 1964) of less than 1 year; contrarily, some 70% of the cases had a registered employment of 5 years or more. Only cancer cases were included; thus, in the absence of population denominators, a proportional risk analysis was performed. The risk for developing cancer at a given site was estimated as the Standardized Proportional Incidence Ratio (SPIR), which is a measure of the proportion of the defined cancer in the companies relative to the proportion of that type of cancer among all employees in Denmark, after adjustment for differences in the distribution of cases over age groups (five years) and calendar periods (one year). The SPIR value approximates the conventional incidence ratio (SIR) when the cancer under investigation constitutes a minor fraction of all malignancies included in the study, when the exposure has no effect on cancer risk in general, and when the overall cancer risk in the cohort is equal to the overall cancer rate in the comparison population. Exact confidence intervals (95% CI) were calculated, assuming a Poisson distribution for the observed frequency (Rothman & Boice, 1979). Tumours were classified according to the Seventh Revision of the International Classification of Diseases (Danish Cancer Registry, 1983).

### Results

Altogether, 398 cases of cancer were observed. Table II gives the distribution of cases according to tumour site, the expected distribution on the basis of all cancers among male

**Table II** Cancer risk, 1970–1984, among males with longest employment in feed processing companies in Denmark between 1964 and cancer diagnosis

| *Site* | *Obs.* | *Exp.* | *SPIR* | *95% CI*[a] |
|---|---|---|---|---|
| (i) *Sites of interest* a priori | | | | |
| Digestive organs | 94 | 94.5 | 99 | 80–122 |
| Liver | 6 | 4.2 | 141 | 57–293 |
| Gallbladder and extra-hepatic bile ducts | 6[b] | 2.7 | 219 | 89–455 |
| Pancreas | 16 | 13.5 | 118 | 70–188 |
| Respiratory system | 82 | 96.8 | 85 | 67–105 |
| Nasal cavities and sinuses | 2 | 1.1 | 176 | 29–580 |
| Larynx | 10 | 7.5 | 133 | 67–237 |
| Lung | 63 | 85.3 | 74 | 58–95 |
| Pleural mesothelioma | 2 | 1.5 | 138 | 23–455 |
| Mediastinum | 5 | 1.4 | 347 | 127–770 |
| (ii) *Other sites* | | | | |
| Buccal cavity and pharynx | 12 | 11.6 | 103 | 56–176 |
| Salivary glands | 4 | 0.8 | 480 | 152–1157 |
| Male genital organs | 37 | 41.4 | 89 | 63–123 |
| Urinary system | 46 | 48.1 | 96 | 70–128 |
| Skin | 58 | 53.5 | 108 | 82–140 |
| Lymphatic and haemato-poietic tissues | 34 | 24.5 | 139 | 96–194 |
| Multiple myeloma | 7 | 3.6 | 196 | 86–388 |
| Other specified sites | 21 | 15.2 | 138 | 86–211 |
| Thyroid | 4 | 1.3 | 308 | 98–742 |
| Endocrine glands | 2 | 0.6 | 342 | 57–1130 |
| Secondary and unspecified sites | 12 | 11.4 | 105 | 57–179 |

[a]Due to multiple comparisons (45 subgroups of malignamcies are tested) approximately two 'significant' results were expected. [b]Includes one incorrectly classified intrahepatic cholangiocarcinoma (see **Table IV**).

employees, the corresponding risk estimates, and the 95% CI. The table is split up into sites of interest *a priori* (liver, biliary tract and respiratory system) and 'other sites'. A nonsignificant, 1.4-fold increase in risk for primary liver cancer and a marginally significant, 2.2-fold increase in risk for cancer of the gall bladder and extrahepatic bile ducts emerged, with 12 cases observed compared to 7.0 expected for the two sites combined. Strikingly, the risk for lung cancer was significantly decreased, with 63 cases observed compared to 85.3 expected (SPIR = 74). Moreover, Table II shows that significant increases in relative risk were observed for cancers of the salivary glands (SPIR = 480) and of thyroid gland (SPIR = 308), with four cases of each observed. A nonsignificantly increased risk was found for cancers of other endocrine glands; however, this observation was based on only two cases observed *versus* 0.6 expected (SPIR = 342). Cancers of the major glands of the body thus accounted for a total of 26 cases observed compared to 16.2 expected (SPIR = 161; 95% CI = 107–232); after exclusions of cases of pancreatic cancer, there were 10 cases observed versus 2.7 expected (SPIR = 370; 95% CI = 188–660). The table also shows a marginally significant increased risk for multiple myeloma (SPIR = 196).

Table III gives the risks for cancer at selected sites in patients whose employment at one of the companies started at least 10 years prior to tumour diagnosis. Owing to this restriction the total number of cancer cases was lower; however, in some subgroups more cases were observed since restriction of the employment period changed the classification of longest-held employment for some cases. All but 7 patients included in this latency period analysis had had a registered length of employment of at least one year. The risk for liver cancer increased substantially under these conditions, with 7 cases observed compared to only 2.8 expected (SPIR = 246; 95% CI = 108–486). The risk for cancers of the gall bladder and extrahepatic bile ducts was also increased (SPIR = 298), yielding a total of 12 cases of cancer of the liver and bilary tract *versus* 4.5 expected. Table IV gives additional data on the cases of liver and biliary-tract cancers. Four cases were not confirmed histopathologically, and one case of cholangiocarcinoma (case no. 8) classifed as a cancer of the biliary tract was in fact intrahepatic. One of the liver cancer cases was observed in a 38 year-old man (case no. 7).

Although based on small numbers, Table III shows that the risk for cancer of the salivary and endocrine glands, the latter defined by ICD-7 = 195, remained high among the employees after introduction of the 10-year latency, while the risk for thyroid cancer was reduced and the risk for pancreatic cancer was eliminated. Similarly, the excess risk for non-Hodgkin's lymphoma disappeared after the introduction of the latency, while the risk for chronic lymphatic leukaemia remained high, although non-significant. The risk for multiple myeloma increased to a marginally significant value of 238, but was still non-significantly elevated. The risk for lung cancer remained below unity (SPIR = 84; 95% CI = 62–112).

For comparison, Table V gives the risks for liver cancer, biliary tract cancer and lung cancer among male employees in (1) farming, forestry and fishing, (2) manufacturing, (3) transport, storage and communication, and (4) wholesale, the latter group including employees in the feed processing industries. This information was derived from the same linked data set used as the basis for the present analysis.

### Discussion

A positive correlation has been reported between estimated

**Table III** Risks for cancer, 1970–1984, at selected sites among males with longest employment in feed processing companies in Denmark

| | *Employment held longest* | | | | |
|---|---|---|---|---|---|
| | *Ever* | | *Ten or more years before diagnosis* | | |
| *SITE (ICD-7 code)* | *O/E* | *SPIR* | *O/E* | *SPIR* | *95% CI* |
| Liver (155.0) | 6/ 4.3 | 164 | 7/ 2.8 | 246[a] | 108– 486 |
| Gall bladder and extrapehatic bile ducts (155.1) | 6[b]/ 2.7 | 219 | 5[b]/ 1.7 | 298[a] | 109– 659 |
| Pancreas (157) | 16/13.5 | 118 | 9/ 8.4 | 108 | 52– 197 |
| Salivary glands (142) | 4/ 0.8 | 480[a] | 2/ 0.5 | 428 | 72–1415 |
| Thyroid (194) | 4/ 1.3 | 308[a] | 1/ 0.7 | 139 | 7– 687 |
| Endocrine glands (195) | 2/ 0.6 | 342 | 1/ 0.3 | 292 | 15–1442 |
| Non-Hodgkin's lymphoma (200, 202) | 11/ 7.4 | 150 | 4/ 4.6 | 87 | 28– 211 |
| Chronic lymphatic leukaemia (204, partly) | 6/ 4.1 | 148 | 4/ 2.5 | 158 | 50– 382 |
| Multiple myeloma (203) | 7/ 3.6 | 196 | 5/ 2.1 | 238 | 87– 528 |
| Lung (162.0, 162.1) | 63/85.3 | 74[a] | 45/53.4 | 84 | 62– 112 |

[a]$P < 0.05$. [b]Includes one incorrectly classified intrapehatic cholangiocarcinoma (see **Table IV**).

**Table IV** Cases of cancer of the liver and the biliary tract, with tumour histopathology when available

| *No.* | *Age (years)* | *Site* | *Histopathology* |
|---|---|---|---|
| 1 | 76 | Liver | Hepatocarcinoma |
| 2 | 77 | Liver | |
| 3 | 72 | Liver | Hepatocarcinoma |
| 4 | 70 | Liver | |
| 5 | 74 | Liver | Adenocarcinoma |
| 6 | 55 | Liver | Hepatocarcinoma |
| 7 | 38 | Liver | Hepatocarcinoma |
| 8 | 53 | Liver[a] | Cholangiocarcinoma |
| 9 | 59 | Gall bladder | Cholangiocarcinoma |
| 10 | 63 | Extrahepatic bile duct | |
| 11 | 62 | Gall bladder | Adenocarcinoma |
| 12 | 55 | Ampulla of Vater | |
| 13 | 80 | Bile duct | Adenocarcinoma |

[a]Incorrectly classified as a biliary-tract cancer.

**Table V** Risks for cancer at selected sites among males with longest-held employment in farming, manufacturing, transportation and wholesale (males in feed processing companies included in the latter)

| Industry | Liver | | Biliary tract | | Lung | |
|---|---|---|---|---|---|---|
| | *O/E* | *SPIR* | *O/E* | *SPIR* | *O/E* | *SPIR* |
| Agriculture, forestry and fishing | 5/ 18.4 | 27[a] | 13/ 11.7 | 111 | 307/ 361.1 | 85[a] |
| Manufacturing | 246/236.8 | 104 | 141/151.8 | 93 | 4975/4695.8 | 106[a] |
| Transport, storage and communication | 80/ 71.5 | 112 | 32/ 45.3 | 71[a] | 1516/1412.3 | 107[a] |
| Wholesale | 75/ 67.3 | 112 | 46/ 43.5 | 106 | 1247/1334.9 | 93[a] |

[a]$P<0.05$.

levels of aflatoxin intake and the incidence of primary liver cancer in several populations (Peers & Linsell, 1977; Van Rensburg *et al.*, 1985; Peers *et al.*, 1987), but only a few studies have been carried out in which the risk for liver cancer has been linked to estimated individual intake (Bulatao-Jayme *et al.*, 1982; Autrup *et al.*, 1987).

In this analysis of workers in feed processing companies in Denmark, although the overall risk for cancer of the digestive organs was close to unity, with an estimated SPIR of 99, individual elevated risks were observed for cancers of the liver, biliary tract, pancreas and salivary glands, the latter being significantly in excess of expectancy. An increase in risk for liver and biliary tract cancer was to be expected in this study of aflatoxin exposed employees. Recently, an assessment was undertaken of the risk for liver cancer in the USA associated with ingestion of aflatoxins from peanuts (Dichter, 1984). On the basis of the dose-effect relationship seen for oral consumption of aflatoxins in the US study and of the expected liver cancer incidence in the age-groups represented by the workers (10 per 100.000), one could expect a 2.7-fold increase in risk for liver cancer following a daily exposure to 170 ng aflatoxin, assuming that aflatoxins are carcinogenic to the liver after inhalation as by ingestion. This estimate corresponds closely to the estimated 2.46-fold elevation in risk for liver cancer found in this study after a latency of 10 years or more. The elevated risk for biliary tract cancer may be associated with transportation and storage of the toxins: adenocarcinomas of the gall bladder and bile duct have been observed in at least one study of monkeys exposed to aflatoxins (Sieber *et al.*, 1979).

In contrast to the Dutch study of aflatoxin exposed workers (Hayes *et al.*, 1984), no excess of lung cancer was observed in this study, and, in fact, the risk was found to be decreased to the same magnitude as among males in farming. The deaths due to respiratory cancer reported in the Dutch study were all observed within a period of 11 years after initial exposure to aflatoxins, and no relationship was found between length of exposure or type of work and cancer risk. Risk factors other than oil-press work may be of importance for the reported association. The significantly decreased risk for lung cancer observed in the present study may be explained partly by the fact that the workers in these companies were not permitted to smoke, due to risk of fire. It may also be due partly to a selective recruitment of workers from rural areas, who have a low risk for lung cancer.

As a side result our study also indicates that the risk for cancer at all of the major glands of the body, including the pancreas, may be increased. However, the risks for cancers of the thyroid and pancreas disappeared after including the 10 year latency period, and the risk for cancer at other endocrine glands was reduced, showing that the relationship with exposure to aflatoxins is not causal. Only the risk for salivary gland cancer remained increased, but the observed numbers were too small to allow any definite conclusion. An elevated risk for multiple myeloma was observed, with an increasing trend after a 10-year latency. No association with aflatoxins has been reported previously, but an association between risk for multiple myeloma and industrial exposure to grain-dust has been reported recently (Alavanja *et al.*, 1987).

The present study is based on analysis of proportionate cancer occurrence which implies that the validity of the SPIR value depends on the overall cancer risk in the cohort being equal to the overall cancer rate in the comparison population. Since lung cancer, which constitutes approximately one-fifth of all cancers in the male population at large, is suspected to be reduced by some 15–20% among the company workers this affects the risk estimates of other sites in the direction of an overestimation. With risks of other common cancers (Table II) close to unity this overestimation will appear on the average of 5%.

Since the occupational history of the cases before 1 April 1964 could not be ascertained in our study design, the cumultive aflatoxin exposures of individual cancer cases could not be determined, and no attempt was made to group the individual feed processing companies according to dustiness of work places or degree of aflatoxin contamination. No distinction was made between production workers, transport workers and clerks. If anything, this would lead to an unknown degree of attenuation of the relative risks reported, since the employees include persons not exposed to aflatoxin-contaminated dust or exposed to only low levels of the toxins. Such misclassification in case-control studies tends to bias the risk estimate towards unity (Bross, 1984; Blettner & Wahrendorf, 1984).

Other predominent causes of primary liver cancer in Denmark are persistent infection with hepatitis B virus and alcoholism. We have no reason to believe that the prevalence of these two factors is any higher among male employees in feed processing companies than among all male employees in the country. The risk for other alcohol related cancers were not increased: and since the low incidence of lung cancer observed in the study group points to below-average tobacco consumption, there was thus most likely below-average alcohol consumption.

Exposure to aflatoxins in the imported feed is the most probable explanation for our finding of an elevated risk for liver cancer and cancers of the biliary tract in this population. Although the increased risks for salivary gland tumours and multiple myeloma may be due to the same exposures, the result remains to be corroborated. Despite the fact that the employees were exposed to aflatoxins primarily *via* the respiratory tract, a decreased risk for lung cancer was observed; thus, the lung does not appear to be a target organ for the carcinogenic effect of aflatoxins.

This research was supported by grants from the Danish Cancer Society. The measurements of aflatoxin $B_1$ were performed by the Danish Government Foodstuff Control and were kindly released by Th. Kofoed, Director of the institute and Bent T. Viuf, research assistant. The measurements of organic dust in the feed processing industries were made by the Institute of Occupational Health. We would like to thank Dr Ole Møller Jensen, head of the Cancer Registry, for his encouragement and support of this work.

### References

ALAVANJA, M.C.R., RUSH, G.A., STEWART, P. & BLAIR, A. (1987). Proportionate mortality study of workers in the grain industry. *J. Natl Cancer Inst.*, **78**, 247.

AUTRUP, H., SEREMET, T., WAKHISI, J. & WASUNNA, A. (1987). Aflatoxin exposure measured by urinary excretion of aflatoxin $B_1$-guanine adduct and hepatitis B virus infection in areas with different liver cancer incidence in Kenya. *Cancer Res.*, **47**, 3430.

BLETTNER, M. & WAHRENDORF, J. (1984). What does an observed relative risk convey about possible misclassification?. *Meth. Inf. Med.*, **23**, 37.

BROSS, I. (1984). Misclassification in $2 \times 2$ tables. *Biometrics.*, **10**, 478.

BULATAO-JAYME, J., ALMERO, E.M., CASTRO, C.A., JARDELEZA, T.R. & SALAMAT, L.A. (1982). A case-control dietary study of primary liver cancer risk from aflatoxin exposure. *Int. J. Epidemiol.*, **11**, 112.

BURG, W.R., SHOTWELL, O.L. & SALTZMAN, B.E. (1981). Measurements of airborne aflatoxins during the handling of contaminated corn. *Am. Ind. Hyg. Assoc. J.*, **42**, 1.

DANISH BUREAU OF STATISTICS (1931–1986). *Foreign Trade in Denmark. 1930, . . . , 1985*. Copenhagen.

DANISH CANCER REGISTRY (1983). *Cancer Incidence in Denmark 1978, 1979 and 1980*. Copenhagen: Danish Cancer Society.

DEGER, G.E. (1976). Aflatoxin – human colon carcinogenesis?. *Ann. Intern. Med.*, **85**, 204.

DICHTER, C.R. (1984). Risk estimates of liver cancer due to aflatoxin exposure from peanuts and peanut products. *Food Chem. Toxicol.*, **22**, 431.

DICKINS, F., JONES, H.E.H. & WAYNFORTH, H.B. (1966). Oral, subcutaneous and intratracheal administration of carcinogenic lactones and related substances: The intratracheal administration of cigarette tar in the rat. *Br. J. Cancer*, **20**, 134.

DVORACKOVA, I. (1976). Aflatoxin and alveolar cell carcinoma. *Br. Med. J.*, **i**, 691.

GOVERNMENT FEEDSTUFF CONTROL (1979–1987). *Annual Reports for 1978, 1979, 1980, 1981, 1982, 1983, 1984, 1985 and 1986*. Lyngby.

HAYES, R.B., VAN NIEUWENHUIZE, J.P., RAATGEVER, J.W. & KATE, F.J.W. (1984). Aflatoxin exposures in the industrial setting: An epidemiological study of mortality. *Food Chem. Toxicol.*, **22**, 39.

INTERNATIONAL AGENCY OF RESEARCH ON CANCER (1976). *IARC Monographs on the Evaluation of the Carcinogenic Risk of Chemicals to Humans*, Vol. 10, *Some Naturally Occurring Substances*, Lyon.

INTERNATIONAL AGENCY FOR RESEARCH ON CANCER (1982). *IARC Monographs on the Evaluation of the Carcinogenic Risk of Chemicals to Humans*, Suppl. 4, *Chemicals and Industrial Processes Associated with Cancer in Humans* (*IARC Monographs, Vols. 1–29*). Lyon.

McLAUGHLIN, J.K., MALKER, H.S.R., MALKER, B.K. & 5 others (1987). Registry-based analysis of occupational risks for primary liver cancer in Sweden. *Cancer Res.*, **47**, 287.

OLSEN, J.H. & JENSEN, O.M. (1987). Occupation and risk of cancer in Denmark: an analysis of 93,810 cancer cases, 1970–1979. *Scand. J. Work Environ. Health*, **13/Suppl. 1**, 1.

PEERS, F.G. & LINSELL, C.A. (1977). Dietary aflatoxin and human primary liver cancer. *Ann. Nutr. Aliment*, **31**, 1005.

PEERS, F., BOSCH, X., KALDOR, J., LINSELL, A. & PLUIJMEN, M. (1987). Aflatoxin exposure, hepatitis B virus infection and liver cancer in Swaziland. *Int. J. Cancer*, **39**, 545.

ROTHMAN, K.J. & BOICE, J.D. (1979). *Epidemiologic Analysis with a Programmable Calculator* (NIH publication No. 79-1649). National Institute of Health: Washington, DC.

SIEBER, S.M., CORREA, P., DALGARD, D.W. & ADAMSON, R.H. (1979). Induction of osteogenic sarcomas and tumours of the hepatobiliary system in nonhuman primates with aflatoxin $B_1$. *Cancer Res.*, **39**, 4545.

SORENSEN, W.G., SIMPSON, J.P., PEACH, I.I.I., THEDELL, T.D. & OLENCHOCK, S.A. (1981). Aflatoxin in respirable corn dust particles. *J. Toxicol. Environ. Health*, **7**, 669.

VAN RENSBURG, S.J., COOK-MOZAFFARI, P., VAN SCHALKWYK, D.J., VAN DER WATT, J.J., VINCENT, T.J. & PURCHASE, I.F. (1985). Hepatocellular carcinoma and dietary aflatoxin in Mozambique and Transkei. *Br. J. Cancer.*, **51**, 713.

VISKUM, S. & MADSEN, F. (1985). Aflatoxin in the occupational environment. *Ugeskr. Laeg.*, **147**, 4037. (Orig. Danish).